# Phytochemical-Loaded Biodegradable Nanoemulsions for Eradication of Fungal Biofilms

**DOI:** 10.3390/nano16100574

**Published:** 2026-05-07

**Authors:** Muhammad Aamir Hassan, Harini Chandrababu, Jungmi Park, Vincent M. Rotello

**Affiliations:** Department of Chemistry, University of Massachusetts Amherst, 710 North Pleasant Street, Amherst, MA 01003, USA; muhammadaami@umass.edu (M.A.H.); hchandrababu@umass.edu (H.C.); jungmipark@umass.edu (J.P.)

**Keywords:** fungal infection, essential oil, nanoemulsion, *Candida albicans*, *Candida duobushaemulonii*, *Candida krusei*

## Abstract

Fungal infections are an escalating health threat, especially in hard-to-treat biofilm-associated infections. *Candida* species are the most widespread drivers of wound biofilm and biomedical device-associated infections. In this study, biodegradable nanoemulsions (BNEs) were fabricated by encapsulating active components of three different essential oils—carvacrol (C-BNE), geraniol (G-BNE), and eugenol (E-BNE)—in a polymeric scaffold with a biodegradable crosslinker. The antibiofilm efficacy of BNEs was assessed against 2-day-old biofilms of multiple *Candida* species. C-BNE showed maximum effectiveness against all fungal biofilms as compared to G-BNE and E-BNE. Confocal microscopy further demonstrated that C-BNE efficiently penetrated the biofilm and killed the fungal cells by compromising cell membrane integrity. Overall, this study highlights the potential of essential oil-loaded nanoemulsions against drug-resistant biofilm-associated fungal infections.

## 1. Introduction

Drug-resistant fungal infections are a rapidly increasing global health concern, with 6.5 million cases yearly caused by invasive fungal infections and an estimated mortality rate of ~2.5 million worldwide [1,2]. Among fungal pathogens, *Candida* species are the leading cause of hospital-associated infections globally, with *Candida albicans* being the most prevalent, followed by *Candida krusei* and *Candida duobushaemulonii* [3,4,5,6]. *Candida* species are heteromorphic, capable of existing in both pathogenic filamentous and unicellular yeast forms [7]. They cause a variety of infections, ranging from superficial mucosal infections to life-threatening conditions, including invasive candidemia and bloodstream infections [8].

In superficial infections, *Candida* spp. can colonize mucosal epithelial surfaces or indwelling biomedical devices [9]. Through morphological transitions, these pathogens excrete a biofilm composed of a complex microenvironment of yeast and hyphal cells embedded in extracellular polymeric substances (EPS), which contribute to recurrent infections and, in severe cases, organ failure and death [10].

*Candida* species form complex, dense biofilms that are challenging to treat [11]. The biofilm matrix acts as a protective barrier, shielding fungal cells from environmental fluctuations, sequestering antifungal drugs, and impairing host immune responses [12]. This leads to worse clinical outcomes and promotes the development of drug resistance [13,14]. Multiple classes of antifungal drugs, such as azoles, polyenes, echinocandins, and allylamines, are currently used to treat candidal infections [15]. These antifungal drugs are effective against planktonic fungal cells but less effective against biofilms due to limited penetration into the EPS matrix [16].

Essential oils are bioactive compounds derived from plant extracts that can be used as antifungal agents with minimal toxicity [17]. However, their antimicrobial potential against fungal biofilms is constrained by poor water solubility and hydrophobicity, which limit penetration into the highly polar biofilm matrix [18]. Polymer-based nanocarrier platforms offer a therapeutic delivery strategy for hydrophobic cargos and can efficiently transport them across the biofilm matrix [19]. Polymeric nanoemulsions incorporating essential oils have been used to treat drug-resistant fungal biofilm infections with minimal cytotoxicity to mammalian cells [20,21].

**Figure 1:**
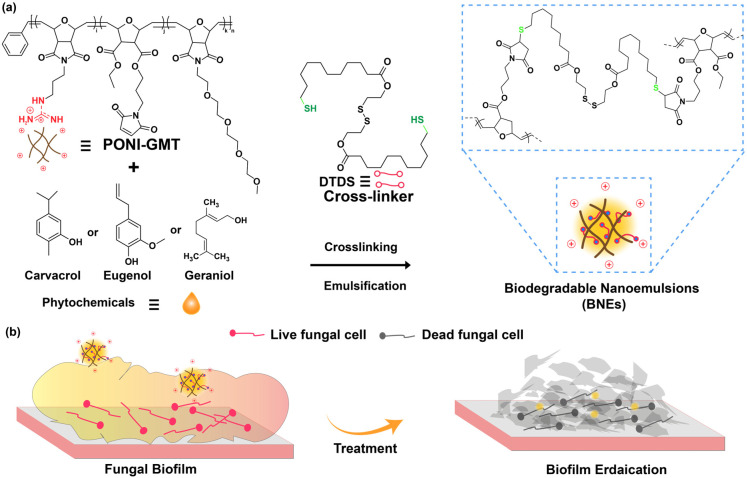
Schematic illustration of essential oil-loaded biodegradable nanoemulsions (BNEs) fabrication and fungal biofilm eradication. (**a**) Emulsification and stabilization of BNEs through dithiol–disulfide (DTDS) crosslinker. (**b**) Penetration of BNE into fungal biofilm and consequent eradication.

Previous studies have employed poly(oxanorborneneimide) (PONI)-based nanoemulsion systems for the delivery of hydrophobic antimicrobials across the EPS matrix of biofilms [22,23]. The PONI scaffolds were functionalized with guanidium, maleimide, and tetraethylene glycol monomethyl ether (PONI-GMT) sidechains that were then crosslinked with a dithiol–disulfide (DTDS) linker to fabricate biodegradable essential oil nanoemulsions (BNEs) (Figure 1). These BNEs featured efficient bacterial biofilm penetration and eradication [24,25]. In the current study, we fabricated essential oil-loaded BNEs using PONI-GMT with DTDS crosslinker. The guanidium group promotes biofilm penetration, while maleimide contributes towards crosslinking, and tetraethylene glycol components enhance the biocompatibility and amphiphilicity of essential oils [26]. The DTDS crosslinker was designed to be biodegradable through interactions with endogenous glutathione and esterase enzymes [24].

We incorporated active compounds of three different essential oils for the fabrication of BNEs, including carvacrol (C-BNE), eugenol (E-BNE), and geraniol (G-BNE), and evaluated their efficacy against different species of *Candida* biofilms. The antibiofilm efficacy of BNEs was evaluated using colony counts and confocal laser scanning microscopy (CLSM) to assess biofilm penetration and eradication. C-BNE demonstrated the highest activity against all tested fungal strains, as compared to G-BNE and E-BNE. Colony counting further validated the antibiofilm efficacy, showing the strongest activity for C-BNE, followed by G-BNE and E-BNE. CLSM Z-stack analysis further demonstrated that C-BNEs efficiently penetrated and eradicated the biofilms using a live/dead staining assay. In addition, the cytotoxicity of BNEs was evaluated in fibroblasts and macrophages, which showed that effective concentrations of the BNEs against fungal biofilms did not compromise mammalian cell viability. Overall, the biodegradable polymeric nanoemulsion platform highlights the potential of essential oils for the treatment of fungal biofilm-associated infections with minimal toxicity.

## 2. Materials and Methods

Fungal strains used in this study include *Candida krusei* [(AR-0397) *Pichia kudriavzevii*] and *Candida duobushaemulonii* (AR-0391), both isolated from blood and obtained from the Antimicrobial Resistance Isolates (ARI, Atlanta, GA, USA) Bank at the Centers for Disease Control and Prevention (CDC). *Candida albicans* (IDRL-7034: isolated from pelvic abscess) was received from the Infectious Diseases Research Laboratory (IDRL), Mayo Clinic, Rochester, MN, USA. Essential oils, including carvacrol (499-75-2), eugenol (97-53-0), and geraniol (106-24-1), were purchased from Millipore Sigma (St. Louis, MO, USA). American Type Culture Collection (ATCC) of NIH/3T3 fibroblast cells (ATCC CRL-1658; ATCC, Manassas, VA, USA) were purchased and cultured in Dulbecco’s Modified Eagle’s Medium (DMEM; ATCC 30-2002, ATCC, Manassas, VA, USA) with 10% fetal bovine serum (SH3007103) from Thermo Fisher Scientific (Waltham, MA, USA). AlamarBlue Cell Viability Reagent (DAL-1100) was used for this study as per the manufacturer’s (Thermo Fisher Scientific, Waltham, MA, USA) instructions.

### 2.1. Fabrication and Characterization of Essential Oil-Loaded Biodegradable Nanoemulsions (BNEs)

Essential oil-loaded biodegradable nanoemulsions (BNEs) were fabricated by emulsification of carvacrol, eugenol, and geraniol with poly(oxanorborneneimide) (PONI-GMT) using dithiol–disulfide (DTDS) crosslinker. Briefly, 3 µL of each oil containing 3 wt% of DTDS crosslinker was mixed with 6 µM of PONI GMT with a final volume of 500 µL. Then, emulsification was performed with an amalgamator (Zoneray HL-AH G8; Hangzhou Zhongrun Medical Instrument Co., Ltd., Hangzhou, China) for 50 s. After fabrication, dynamic light scattering (DLS) was employed to quantify the hydrodynamic diameter of BNEs, and the surface charge of BNEs was measured by zeta potential.

### 2.2. Minimal Inhibitory Concentration (MICs) of BNEs

Single colonies of each fungus were inoculated into 4–5 mL of tryptic soy broth [(TSB): (DF0370-17-3)] supplemented with 1% glucose for the preparation of an overnight culture. For cultivation of mid-log phase culture, 100 µL of overnight-grown culture was inoculated into fresh TSB with 1% glucose and incubated at 30 °C with 220 rpm shaking. Fungal cells were collected by centrifugation for 5 min at 4000 rpm and washed three times with 0.85% NaCl solution. Next, the fungal pellet was resuspended in phosphate-buffered saline [PBS (Gibco: 14-190-144)] and the optical density (O.D) was measured at 600 nm. Serial dilutions of each BNE were prepared in M9 minimal salts (A1374401) with TSB + glucose (9:1) solution in 50 µL. Each dilution of BNEs was mixed with 50 µL of M9 with TSB + glucose containing 1 × 10^6^ CFU/mL of each fungal strain per well. The plate was incubated at 37 °C with 220 rpm shaking overnight. The following day, MIC values of each BNE were analyzed by taking the O.D. at 600 nm. For the time-dependent kill assay, MIC values of BNEs were incubated with fungal cells, and the O.D. was recorded at 600 nm every 1 h for 18 h. The release profile of BNE was measured by using Nile red-labeled C-BNE incubated with 1 × 10^7^ CFU/mL of *C. albicans,* along with the following controls, including Nile red-labeled carvacrol oil only and Nile red-labeled carvacrol oil in water. The fluorescence was recorded every 20 min (Ex: 560 nm and Em: 640 nm).

### 2.3. BNEs Biofilm Penetration Analysis

A mid-log phase culture was harvested, and 1 × 10^8^ CFU/mL of *C. albicans* were seeded into confocal chambers (LAB-TEK:155382) in M9: TSB + glucose media for 48 h. After cultivation, the biofilm was rinsed with PBS three times and treated with C-BNE solution for 3 h. The treated biofilm was washed with PBS three times and stained with 10 μM SYTO 9 and 1.65 μM propidium iodide (PI) for 1 h as per manufacturer’s protocols (Thermo Fisher: L7007). Upon removing the staining solution, the biofilm was washed with PBS and visualized using confocal laser scanning microscopy (CLSM with a TRITC (red) and FITC (green) channels using a Nikon A1 resonant scanning module (Nikon Instruments Inc., Tokyo, Japan). NIS-Element software (Nikon version 5.21.00; Nikon, Tokyo, Japan) was used to process images and Z stacks.

### 2.4. Evaluation of Antibiofilm Efficacy of BNEs

For the identification of the minimal biofilm inhibitory concentration (MBIC) of BNEs, a fungal culture of 1 × 10^8^ CFU/mL was seeded into a 96-well microtiter plate with a pegged lid and incubated for 6 h at 80 RPM on 30 °C. The following peg lid was washed with PBS and dipped into a new 96-well microtiter plate containing serially diluted BNEs and incubated overnight at 37 °C. After incubation, the O.D600 was recorded, and the peg lid was washed with PBS and dipped again into a new 96-well microtiter plate having M9: TSB + glucose media only for overnight to assess the minimal biofilm elimination concentration (MBEC) of BNEs. Next, a 2-day-old biofilm of each fungal strain was cultivated by seeding 100 µL of 1 × 10^8^ CFU/mL into a 96-well microtiter plate (Falcon: CLS351172, Sigma Aldrich, St. Louis, MO, USA) along with media as a negative control. Before treatment, biofilms were rinsed with 100 µL of PBS three times. The fungal biofilms were then treated with serially diluted concentrations of BNEs and incubated for 3 h at 37 °C. Following the treatment, the biofilm was washed with PBS to remove the BNEs, and biofilm viability was evaluated through AlamarBlue assay as per the manufacturer’s protocol (Invitrogen: DAL1025, Invitrogen, Carlsbad, CA, USA). A 110 µL of 1X reagent was added to each well of the 96-well microtiter plate and incubated at 37 °C for 1–2 h. After incubation with treated biofilm, fluorescence intensity was quantified by measuring the reagent solution at excitation and emission wavelengths of 560 nm and 590 nm, respectively. Biofilm viability was further validated by colony counting; treated biofilms were washed and dispersed into PBS and serially spread on Sabouraud Dextrose Agar [(SDA) (Millipore Sigma: 89579-500G-F, St. Louis, MO, USA)] plates. Each treatment was performed in triplicate across two independent experiments.

### 2.5. Multimodal Mechanistic Evaluation of BNE

For the evaluation of cytoplasmic cell membrane depolarization, a mid-log phase culture of C. albicans was harvested, and the cells were washed three times with HEPES buffer (5 mM, 20 mM glucose, pH = 7.2) three times. Fungal cells were treated with 50 nM 3,3′-Dipropylthiadicarbocyanine Iodide (DiSC3(5)) dye for 30 min at 30 °C, followed by the addition of 10 mM KCl. The fluorescence was then recorded at excitation and emission wavelengths of 622 nm and 670 nm, respectively. Once a stable fluorescence baseline was obtained, BNEs were added and incubated for 30–40 min, and the fluorescence was recorded again. For the ROS generation assay, fungal cells were stained with 10 µM of 2,7-Dichlorodihydrofluorescein diacetate (DCFH-DA) and incubated for 30 min in the dark. Stained cells were washed three times with PBS to remove excessive dye. Then, stained cells were treated with BNEs for 1 h, and the fluorescence was recorded at excitation and emission wavelengths of 485 nm and 525 nm. Cell membrane disruption was further visualized using CLSM. 1 × 10^7^ CFU/mL of *C. albicans* were treated with Nile red-loaded C-BNE for 3 h and stained with Calcofluor white (Cat# 18909-100ML-F) and SYTO-9 before CLSM imaging.

### 2.6. Cytotoxicity of BNEs to Mammalian Cells

DMEM media supplemented with 1% antibiotic-antimycotic and 10% FBS was used to culture NIH/3T3 fibroblast cells (ATCC CRL-1658) and macrophage cells (RAW 264.7) seeded at a density of 10,000 cells per well in a 96-well microtiter plate (Corning: 3599, Corning, NY, USA). The seeded cells were incubated for 24 h with 5% CO_2_ at 37 °C in a humid environment. After incubation, the cells were washed with PBS and treated with different concentrations of BNEs for 3 h. Following treatment, the cells were rinsed with PBS prior to the AlamarBlue viability evaluation assay for cell viability.

### 2.7. Statistical Analysis

Statistical analysis was performed using one-way analysis of variance (ANOVA) followed by Tukey’s post hoc test. Statistical significance defined as * *p* < 0.05, ** *p* < 0.01, *** *p* < 0.001, and **** *p* < 0.0001; ns = not significant.

## 3. Results and Discussion

### 3.1. Fabrication and Characterization of Essential Oil-Loaded Biodegradable Nanoemulsions (BNEs)

Carvacrol, eugenol, and geraniol are generally recognized as safe (GRAS) compounds and are the active compounds in oregano, clove, and rose oil, respectively. These compounds have been reported to possess antifungal properties with multimodal mechanisms of action, including disruption of fungal cell membranes, quorum quenching, and ROS generation [17,27]. Essential oil-loaded BNEs [carvacrol (C-BNE), eugenol (E-BNE), and geraniol (G-BNE)] were fabricated via emulsification with amphiphilic PONI-GMT polymer using DTDS crosslinker (Figure 1). The guanidium functional group of PONT-GMT provides a cationic charge to BNEs, which facilitates better interaction with the negatively charged EPS matrix of biofilms (Figure 1b). Maleimide contributes to stability by crosslinking with the biodegradable DTDS crosslinker, and the TEG units enhance the solubility of the BNEs payload in aqueous medium (Figure 1a).

**Figure 2:**
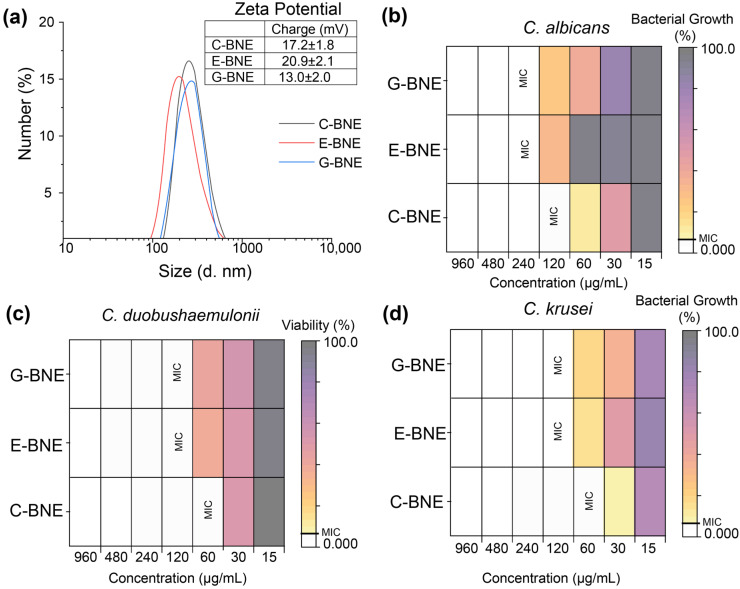
Characterization and minimum inhibitory concentrations (MICs) of BNEs. (**a**) Hydrodynamic size of BNEs measured by dynamic light scattering (DLS) and surface charge determination by zeta potential analysis. Heatmap of MIC values of BNEs against (**b**) *C. albicans*, (**c**) *C. duobushaemulonii*, (**d**) *C. krusei,* where white boxes indicate no detectable fungal growth, while MIC-labeled boxes represent the minimum inhibitory concentration (MIC) for each BNE against the particular species.

After fabrication, the hydrodynamic particle size of BNEs was characterized using DLS, which showed diameters of ~180 nm for E-BNE and ~220 nm for C-BNE and G-BNE. The zeta potential analysis showed a positive surface charge of 20.9 ± 2.1 for E-BNE, ~17.2 ± 1.8 mV for C-BNE, and ~13.0 ± 2.0 mV for G-BNE (Figure 2a), suitable for penetration of the negatively charged EPS matrix of fungal biofilms [28]. Transmission electron microscopy revealed that BNEs have spherical morphology (Figure S3). We have previously reported the stability of BNEs, which form stable assemblies for more than one month, attributed to the DTDS crosslinker [29].

### 3.2. Minimal Inhibitory Concentration (MICs) of BNEs

The MIC analysis of BNEs demonstrated species-specific antifungal efficacy. Overall, *C. albicans* (IDRL-7034) required around a two-fold higher concentration of BNEs as compared to *C. duobushaemulonii* (AR-0391) and *C. krusei* (AR-0397) (Figure 2b–d). C-BNE consistently exhibited the lowest MIC value (60 µg/mL), indicating an almost 2-fold better efficacy than E-BNE and G-BNE (120 µg/mL) against all tested fungi, including *C. albicans*, *C. duobushaemulonii,* and *C. krusei.* A similar MIC pattern was observed against *C. albicans*, where C-BNE showed 2-fold better activity than E-BNE and G-BNE (Figure 2a). However, essential oils alone demonstrated much higher MIC values against all fungi than BNEs, likely due to poor solubility in media. For voriconazole, low MIC values of 7.5 µg/mL were observed against *C. albicans* and *C. krusei*, and 15 µg/mL against *C. duobushaemulonii,* whereas terbinafine showed higher MICs against all tested fungi (Table S1). A time point study also revealed that at a concentration of 120 µg/mL, C-BNE completely inhibited the growth of *C. albicans*, followed by G-BNE, which inhibited growth up to 6 h. In contrast, E-BNE showed lower effectiveness, inhibiting the growth for only 4 h at a similar concentration (Figure S2). Next, the BNE release profile showed that oil was released from crosslinked PONI GMT arrangements after exposure to *C. albicans* in 3 h, as indicated by a gradual decrease in fluorescence due to quenching of Nile red fluorescence in aqueous media (Figure S6).

### 3.3. BNEs Biofilm Penetration Analysis

**Figure 3:**
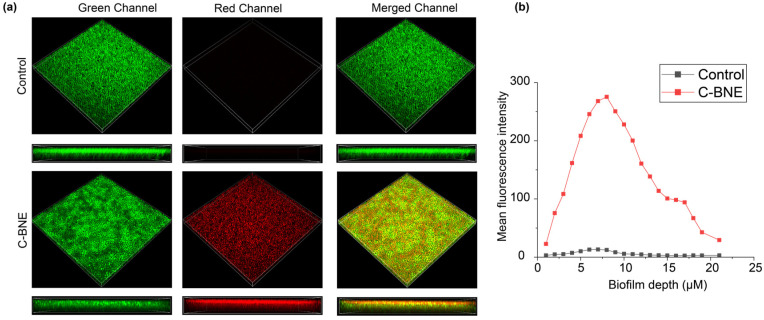
Representative 3D confocal images of 2-day-old *C. albicans* biofilms following 3 h of C-BNE treatment. (**a**) Biofilms were stained with SYTO-9 (green channel) and propidium iodide (PI, red channel) in both C-BNE–treated and untreated control groups. (**b**) Quantification of red fluorescence (propidium iodide) from Z-stack slices obtained from CLSM images after treatment of C-BNE against *C. albicans*.

Biofilm penetration is one of the leading challenges for antifungal agents, including essential oils [30,31]. The guanidinium scaffold of PONI GMT polymer imparts a positive charge, which enhances penetration into the negatively charged matrix of biofilms and improves the stability of essential oils in aqueous media [22,24]. A 2-day-old biofilm of *C. albicans* was treated with 480 µg/mL of C-BNE for 3 h. Following treatment, the biofilm was stained with SYTO-9, which labels live and dead cells in green, and PI, which selectively stains the cells with compromised membrane integrity in red. The confocal image indicated that C-BNE treatment disrupted the *C. albicans* biofilm and targeted fungal cells by compromising their membrane integrity, as evidenced by red fluorescence compared to the untreated control. Furthermore, Z-stack analysis confirmed efficient penetration of C-BNE throughout the biofilm, as visualized in colocalized yellow fluorescence in the side view (Figure 3a). The red fluorescence was also quantified using ImageJ (Fiji, NIH, Bethesda, MD, USA) [32] by analyzing mean fluorescence intensity across each slice of the Z stack, which presented efficient C-BNE penetration and embedded fungal cell eradication (Figure 3b). These results are consistent with previous studies that have reported that amphiphilic polymers with cationic charge efficiently penetrated biofilms [33,34].

### 3.4. Evaluation of Antibiofilm Efficacy of BNEs

**Figure 4:**
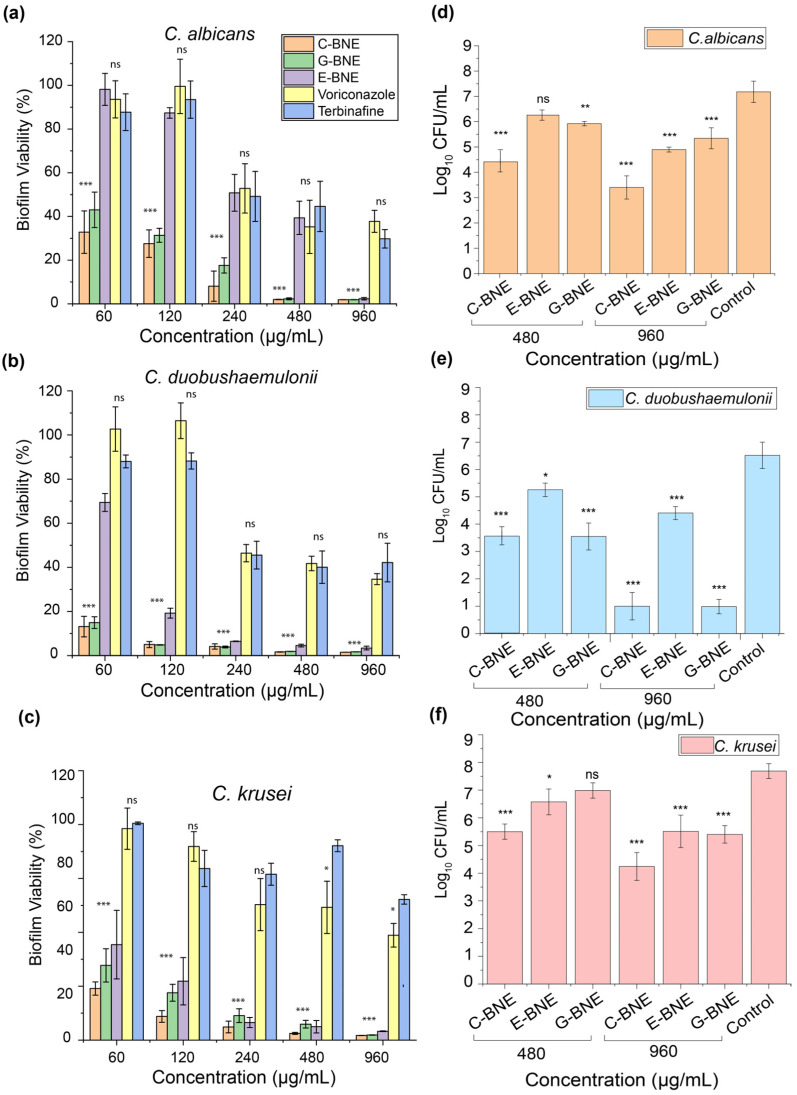
Antibiofilm efficacy of biodegradable nanoemulsions (BNEs) against 2-day-old biofilms and biofilm viability evaluated by AlmarBlue assay of (**a**) *C. albicans*, (**b**) *C. duobushaemuloni*, (**c**) *C. krusei*. Biofilm viability was also assessed by colony counts for (**d**) *C. albicans*, (**e**) *C. duobushaemuloni*, (**f**) *C. krusei*. All the values are expressed as mean ± standard deviation from at least three replicates. Data are expressed as mean ± standard deviation, and Biofilm analysis was conducted using one-way analysis of variance (ANOVA) with statistical significance defined as * *p* < 0.05, ** *p* < 0.01, and *** *p* < 0.001; ns = not significant.

The minimal biofilm inhibitory concentration (MBIC) and minimal biofilm eradication concentration (MBEC) were evaluated against *C. albicans*. C-BNE exhibited MBIC and MBEC values from 120 to 240 µg/mL, representing efficient inhibition and eradication. In comparison, G-BNE and E-BNE demonstrated higher MBIC and MBEC values, 480–960 µg/mL, respectively (Figure S5a,b). The antibiofilm activities of C-BNE, E-BNE, and G-BNE were tested against 2-day-old biofilms of *C. albicans*, *C. duobushaemulonii*, and *C. krusei*. Following a 3 h treatment, fungal biofilm eradication was assessed using the AlamarBlue assay and colony counting method. The AlamarBlue viability analysis revealed that C-BNE reduced fungal cell viability by ~99%, while 2-fold higher concentrations of G-BNE and 4-fold higher concentrations of E-BNE achieved comparable biofilm reductions (Figure 4a). Against *C. duobushaemulonii* and *krusei*, a similar antibiofilm efficacy pattern was observed (Figure 4b,c). We observed lower MIC values of BNEs against planktonic fungal cells, while higher concentrations were required to acquire antibiofilm activity. This discrepancy can be attributed to the heterogeneity of biofilm, composed of a complex microenvironment containing a mixed population of metabolically active and inactive cells required higher concentration of BNEs [33,34].

However, conventional antifungal drugs, including terbinafine and voriconazole, did not demonstrate effective eradication of fungal biofilms even at very high concentrations, i.e., 960 µg/mL, which showed a 50% reduction in biofilm (Figure 4a–c). Antibiofilm efficacy of essential oils alone showed minimal activity against all fungi at the higher concentration of 960 µg/mL (Figure S1a–c). Although the essential oil and antifungal drugs showed lower MIC values, restricted antibiofilm activity is likely due to limited solubility in aqueous media and inadequate penetration into the EPS matrix of biofilm (Figure 4a–c).

Antibiofilm efficacy of BNEs was further validated against 2-day-old biofilms following 3 h of treatment, and viability was evaluated by CFU enumeration. The C-BNE exhibited ~4 log_10_ CFU/mL at a concentration of 960 µg/mL against *C. albicans* and *C. krusei*, and almost complete eradication of *C. duobushaemulonii* biofilm (~6 log_10_ CFU/mL). In comparison, G-BNE showed ~6 log_10_ for *C. duobushaemulonii* and ~2–3 log_10_ for other species, while E-BNE exhibited ~2–3 log_10_ across all species at a similar concentration, indicating comparatively lower activity (Figure 4d–f). The species-specific antibiofilm efficacy of BNEs is likely to differ in the composition, thickness, and architecture of EPS barriers [14]. *C. albicans* has been reported to develop a well-organized and complex architecture of EPS matrix in dense biofilms, whereas *C. krusei* shows a less organized microenvironment [35]. In the case of *C. duobushaemulonii*, the biofilm exhibits a much simpler architecture with a weaker EPS barrier, which likely makes the biofilm more susceptible to BNE treatment [36]. Overall, C-BNE presented 2–4-fold better antibiofilm efficacy against all fungi, followed by G-BNE and E-BNE. Our results showed a significant reduction in biofilms of *C. albicans* and *C krusei*, along with complete eradication of *C. duobushaemulonii* biofilm. These findings are consistent with our previous studies [21]. Previously, PONI GMT-based biodegradable nanoemulsions have been extensively investigated for the eradication of bacterial biofilms [23,25,26]. However, this is the first study to extend the application of the BNEs system against fungal biofilms, which showed efficient penetration and eradication. These results showcase broader antimicrobial applicability across both bacterial and fungal biofilm eradication. Fewer studies have reported the use of nanoemulsions for the eradication of fungal biofilms. Furthermore, this study demonstrates stronger antifungal activity as compared to previous studies on nanoemulsions, which often require higher concentrations (33 µL/mL of nanoemulsion) and present lower antibiofilm inhibition activity (70% reduction) against *C. albicans* [37]. Another study reported that NB-201 nanoemulsion showed ~70% biofilm inhibition at a 1/64 dilution with minimal cytotoxicity toward mammalian cells. However, all the BNEs included in this study demonstrated >90% biofilm at 240 µg/mL reduction without compromising mammalian cell viability [38].

### 3.5. Multimodal Mechanistic Evaluation of BNE

**Figure 5:**
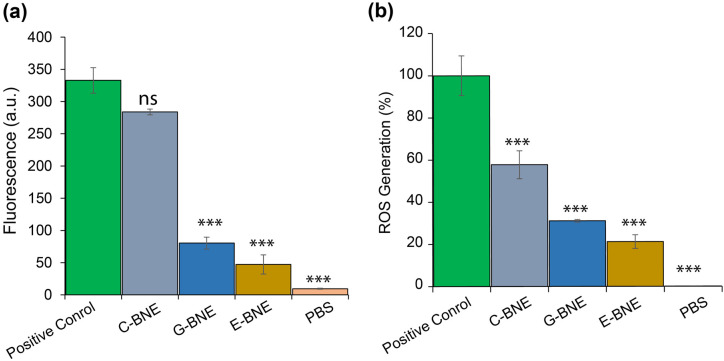
Multimodal mechanism of essential oil-loaded biodegradable nanoemulsion (BNEs) against *C. albicans*. (**a**) Cytoplasmic membrane depolarization with treatment of BNEs. (**b**) ROS generation upon exposure to BNEs. Data are expressed as mean ± standard deviation, and statistical analysis was conducted using one-way analysis of variance (ANOVA) with statistical significance defined as *** *p* < 0.001; ns = not significant.

The cytoplasmic cell membrane leakage and ROS generation of BNEs were evaluated against *C. albicans*. Fungal cells were treated with DiSC3(5) and incubated with BNEs along with a positive control (0.1% Triton X-100) for 1 h, and fluorescence was recorded. The result exhibited cytoplasmic membrane polarization, with C-BNE showing results comparable to the positive control, followed by G-BNE and E-BNE (Figure 5). C-BNE showed cytoplasmic membrane depolarization comparable to 0.1% Triton X, whereas G-BNE and E-BNE exhibited lower levels of membrane depolarization compared to C-BNE. For ROS generation analysis, *C. albicans* cells were stained with DCFH-DA and treated with BNEs along with 1 mM H_2_O_2_. The ROS generation relative to the positive control indicated that C-BNE induced higher ROS generation (60%), followed by G-BNE (35%) and E-BNE (25%). Higher cytosolic membrane depolarization and ROS generation by C-BNE provided superior antimicrobial efficacy compared to G-BNE and E-BNE. Cell membrane disruption was further visualized through CLSM. Nile red-loaded C-BNE (red color) was incubated with *C. albicans* for 3 h, followed by staining with Calcofluor white (blue color) and SYTO-9 (green color). The treated cells were observed using CLSM, which revealed that C-BNE disrupted the cell membrane and facilitated the delivery of the Nile red-labeled C-BNE across the cell membrane (Figure S4). Carvacrol is an active ingredient of oregano oil which showed potential antimicrobial activity via multimodal mechanisms of action, including cell membrane disruption, ROS generation, and quorum quenching, while geraniol and eugenol have been reported with cytosolic leakage and ROS generation [39,40]. The multimodal mechanisms of BNEs’ action offer a potential strategy for the treatment of MDR infections with minimal resistance development against them [21].

### 3.6. Cytotoxicity of BNEs to Mammalian Cells

**Figure 6:**
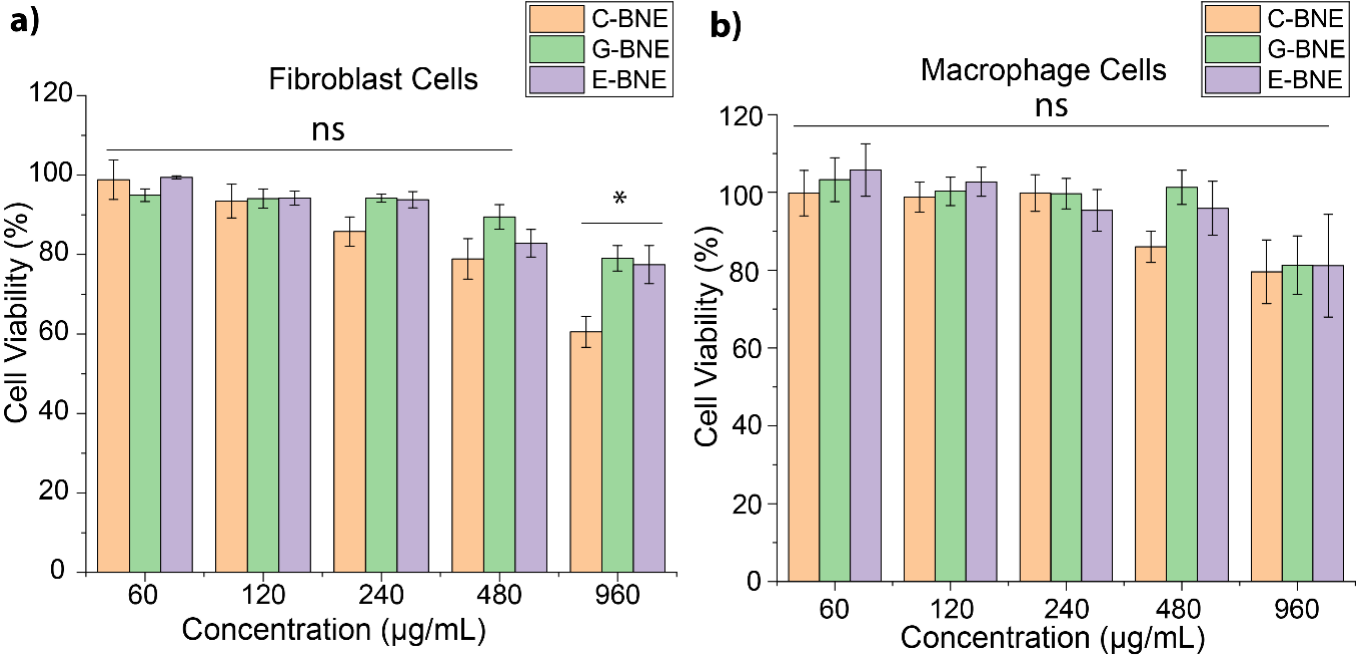
Analysis of BNEs biocompatibility with different mammalian cells. The mammalian cell viability was evaluated through AlamarBlue assay following 3 h exposure to (**a**) Fibroblast cells (ATCC CRL-1658) and (**b**) Macrophage cells (RAW 264.7). All the values are expressed as mean ± standard deviation from at least three replicates. Data are expressed as mean ± standard deviation, and statistical analysis was conducted using one-way analysis of variance (ANOVA) with statistical significance defined as * *p* < 0.05; ns = not significant.

Fibroblast and macrophage cells play an essential role in wound healing and actively interact with fungal biofilms in infected wounds [41,42]. The cytotoxic effect of BNEs on fibroblast cells (ATCC CRL-1658) and macrophage cells (RAW 264.7) was assessed. The cells were treated with different concentrations of BNEs, and cell viability was evaluated after 3 h of treatment by AlamarBlue assay, consistent with biofilm treatment time. The results revealed that there was little or no toxicity at BNE concentrations required to kill fungi (120–480 µg/mL). Even at 950 µg/mL, G-BNE and E-BNE did not compromise viability, and C-BNE showed moderate cytotoxicity to fibroblast cells and macrophage cells (Figure 6). Overall, therapeutic concentrations of C-BNE, G-BNE, and E-BNE showed minimal cytotoxicity on fibroblast and macrophage cells. Thus, the incorporation of essential oil into polymeric nanoemulsion demonstrated effective treatment for biofilm-associated fungal infections with minimal cytotoxicity to mammalian cells without generating resistance [21].

## 4. Conclusions

This investigation demonstrated the ability of polymeric nanoemulsions to combat challenging fungal biofilms. BNEs, especially C-BNE and G-BNE, provide an effective approach for the eradication of fungal biofilms by enhancing the solubility of essential oils in aqueous media and delivering them across the EPS biofilm matrix. Essential oil-loaded BNEs represent a promising approach for combating fungal biofilms with minimal cytotoxicity to mammalian cells. Additionally, the multimodal mechanisms of essential oils make them promising tools for targeting drug-resistant fungal infections. Incorporation of GRAS constituents into the nanoemulsion system further enhances its biosafety profile toward broader application and clinical translation. Furthermore, in vivo application and pre-clinical trials are required to confirm the therapeutic potential of this research. However, this study contributes to advancing efficient solutions for treating fungal biofilm infections.

## Data Availability

The raw data supporting the conclusions of this article will be made available by the authors on request.

## Supplementary Materials

The following supporting information can be downloaded at: https://www.mdpi.com/article/10.3390/nano16100574/s1, Table S1: MIC values of BNEs, antifungal drugs, and essential oils against *C. albicans*, *C. duobushaemulonii*, and *C. krusei*. Figure S1a–c: Antibiofilm activity of essential oils against fungal strains used in this study. Figure S2: Time-dependent killing efficacy of BNEs against *C. albicans*. Figure S3: Representative TEM image of C-BNE nanoparticles. Figure S4: CLSM images of *C. albicans* treated with Nile red-labeled C-BNE, followed by the staining with SYTO-9 and Calcofluor white dye. Figure S5: (a) Minimal biofilm inhibitory concentration (MBIC), (b) minimal biofilm eradication concentration (MBEC) of BNEs against *C. albicans*. Figure S6: Release profile of Nile red-labeled C-BNE following incubation with *C. albicans*.
