# Supplementary material for: Phytochemical-Loaded Biodegradable Nanoemulsions for Eradication of Fungal Biofilms
Source: Nanomaterials (Basel). 2026 May 7;16(10):574. doi: 10.3390/nano16100574 (PMC13209755; doi:10.3390/nano16100574)
Supplement: Supplementary file 1 [file nanomaterials-16-00574-s001.zip › nanomaterials-4220159-supplementary.pdf]

Phytochemical-Loaded Biodegradable Nanoemulsions for Eradication of Fungal Biofilms

Muhammad Aamir Hassan [1], Harini Chandrababu [1], Jungmi Park [1,] and Vincent M. Rotello [1,*]

[1]Department of Chemistry, University of Massachusetts Amherst, 710 North Pleasant Street, Amherst, Massachusetts, 01003, United States
***e-mail**: rotello@chem.umass.edu

Table S1. Minimum inhibitory concentrations (MICs) of BNEs, antifungal drugs, and essential oils against three fungal strains.

MICs(µg/mL)	*C. albicans*	*C. duobushaemulonii*	*C. krusei*
C-BNE	120	60	60
E-BNE	240	120	120
G-BNE	120	120	120
Terbinafine	30	30	60
Voriconazole	7.5	15	7.5
Carvocarol	240	120	120
Eugenol	480	120	240
Geraniol	960	480	960

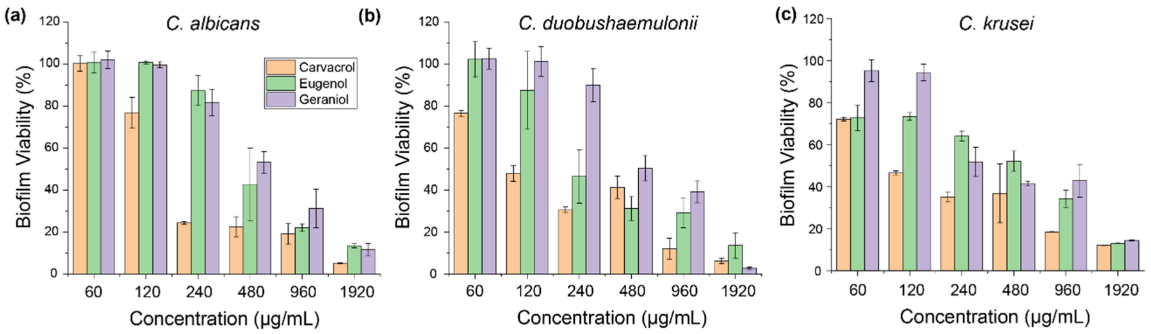

Figure S1. Essential oil antibiofilm activity with 3h treatment against (**a**) *C. albicans* (IDRL-7034), (**b**) *C. duobushaemulonii* (AR-391), (**c**) *C. krusei* (AR-397). Values are presented as mean ± standard deviation from at least three replicates.

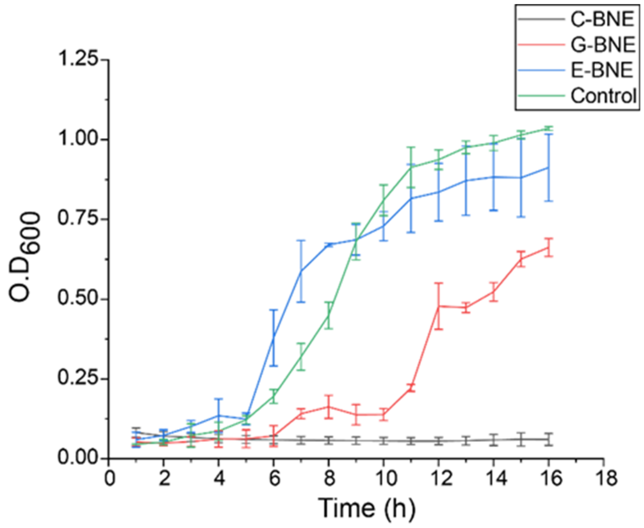

Figure S2. Time-dependent killing efficacy of BNEs against *C. albicans*.

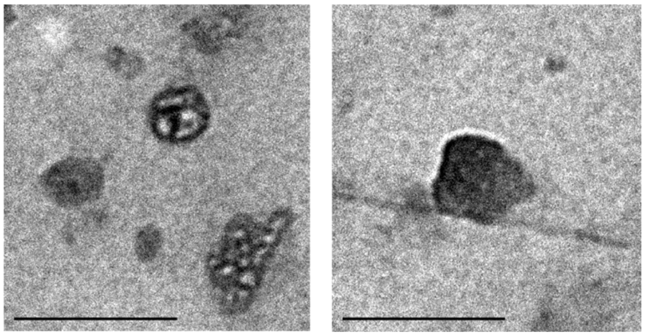

Figure S3. Representative TEM image of C-BNE nanoparticles. The scale bar corresponds to 100 nm.

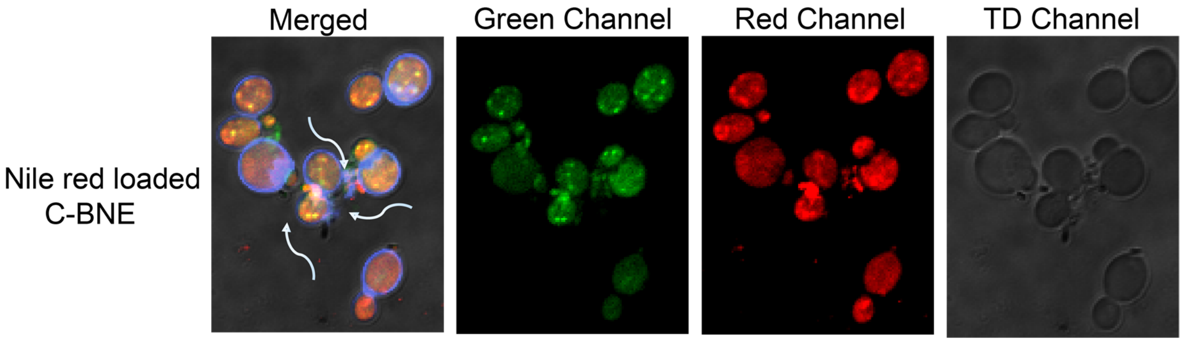

Figure S4. CLSM images of *C. albicans treated* with Nile red-labeled C-BNE, followed by staining with SYTO-9 and Calcofluor white dye.

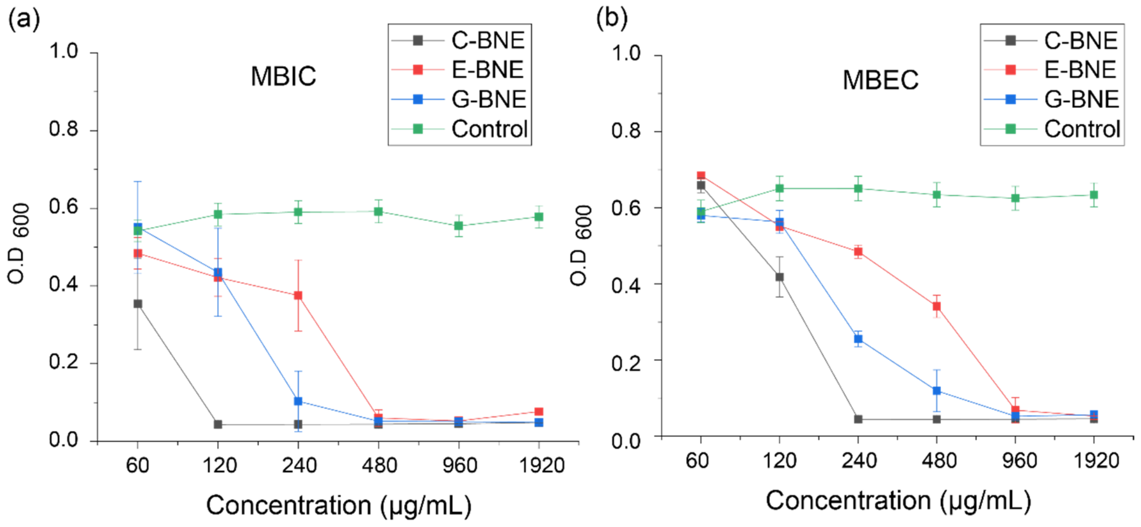

Figure S5. (**a**) Minimal biofilm inhibitory concentration (MBIC), (**b**) minimal biofilm eradication concentrations (MBEC) of BNEs against *C. albicans.*

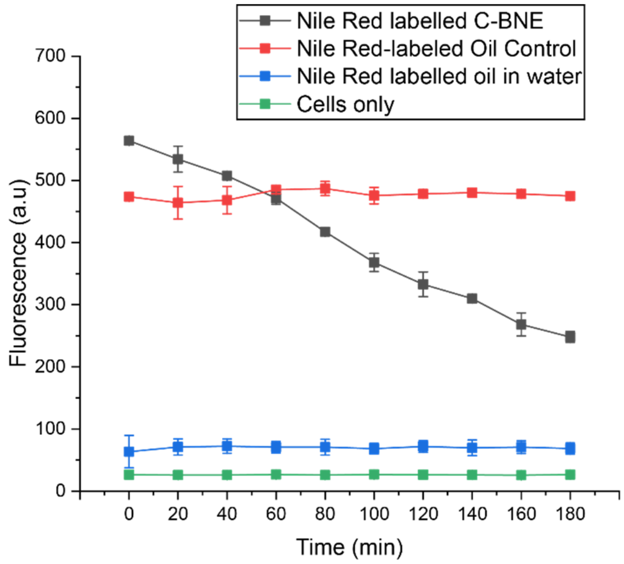

Figure S6. Release profile of Nile red labelled C-BNE following incubation with *C. albicans.*
